# Nutritional Value and Antimicrobial Activity of *Pittosporum angustifolium* (Gumby Gumby), an Australian Indigenous Plant

**DOI:** 10.3390/foods9070887

**Published:** 2020-07-06

**Authors:** Anh Dao Thi Phan, Mridusmita Chaliha, Hung Trieu Hong, Ujang Tinggi, Michael E. Netzel, Yasmina Sultanbawa

**Affiliations:** 1 ARC Industrial Transformation Training Centre for Uniquely Australian Foods, Centre for Nutrition and Food Sciences, Queensland Alliance for Agriculture and Food Innovation, The University of Queensland, Coopers Plans 4108, Australia; a.phan1@uq.edu.au (A.D.T.P.); m.chaliha@uq.edu.au (M.C.); h.trieu@uq.edu.au (H.T.H.); 2 Department of Food Technology, Faculty of Agriculture, Can Tho University, Can Tho 94000, Vietnam; 3 Health Support Queensland, Queensland Health Department, Coopers Plans 4108, Australia; Ujang.Tinggi@health.qld.gov.au

**Keywords:** Gumby Gumby, Australian indigenous plant, nutrients, bioactive compounds, antimicrobial activity

## Abstract

The indigenous endemic plant *P. angustifolium* has received attention for nutraceutical and therapeutic applications in Australia. This study investigates for the first time the nutritional value (macro- and micronutrients, minerals, trace elements, polyphenols, carotenoids, saponins and antioxidant capacity) and antimicrobial activity of different botanical parts of *P. angustifolium*, either collected from the wild or cultivated. Different botanical tissues, geographic location and growing condition (wild vs. cultivated) showed significant (*p* < 0.05) effects on the tested bioactive compounds, with the leaves having significantly (*p* < 0.05) higher levels than the stems. Saponins and polyphenols could be identified as the main bioactive compounds in the leaves with up to 4% per dry weight. The extracts of *P. angustifolium* leaves and stems showed strong antioxidant and antimicrobial activities, especially against *Candida albicans*. These activities correlated (R^2^ = 0.64–0.92; *p* < 0.05) with the levels of polyphenols and saponins, indicating their biologic potential. Findings from this study may provide information for future applications of *P. angustifolium* in the functional ingredient or nutraceutical industry.

## 1. Introduction

There is a global demand for natural ingredients from plant sources with multifunctional properties for application in the food and nutraceutical industries. In 2018, the market for herbal dietary supplements in the United States increased by 9.4% and the value of this market reached an estimated 8.842 billion dollars across all market channels, marking the strongest US sale growth of herbal supplements since 1998 [1]. Similarly, there is a growing demand for foods and medicines that are known for their customary usage by Indigenous communities. These interests range from general desires for prevention from acute and chronic diseases, maintenance of good health, well-being, immune system strengthening and energy sustenance, to seeking out traditional foods/native plants that provide natural antioxidants and antimicrobials. There are many plant bioactive compounds of particular interest such as saponins, polyphenols including tannins and alkaloids, which are known to assist in providing the above benefits to human health and well-being.

*P. angustifolium* (common name: Gumby Gumby) is a shrub tree, native to Australia. This species belongs to the genus Pittosporum and the family Pittosporaceae, consisting of approximately 200 species in nine genera [2]. *P. angustifolium* has been found mainly in inland Australia, New Zealand and many other parts of the world [2]. Different botanical tissues of *P. angustifolium* have been traditionally used as Indigenous bush medicine across inland Australia for hundreds of years to enhance general health and well-being. The infusions from leaves were used to treat cold and coughs and to induce lactation [3]. Decoction made from the fruits was taken orally or applied to treat skin problems such as eczema and pruritus [2,4]. In addition, *P. angustifolium* has been traditionally used for treatment of rheumatoid arthritis and other inflammatory conditions [5]. Recently, Madikizela and McGaw [6] summarized information on traditional medicinal applications of the genus Pittosporum for treatment of a wide range of infections such as inflammatory, spasmodic, malarial and microbial infections (e.g., narcotics, chronic bronchitis, leprous infection, rheumatic, bruises, sciatica, chest infection and certain skin diseases). Interestingly, all parts of the Pittosporum plants, including leaf, bark, root, flower, fruit pulp, seed and even wood, have been reported to show potential medicinal applications in many countries such as Australia, China, India and South Africa [6].

**Figure 1:**
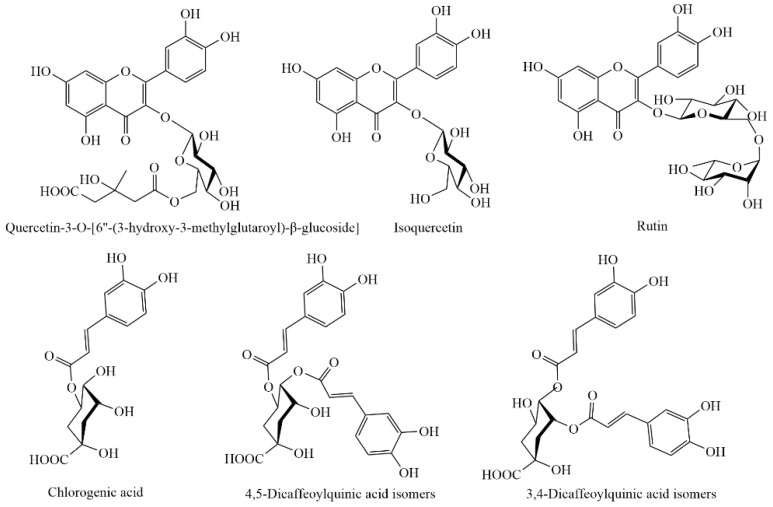
Main polyphenolic compounds identified in *P. angustifolium* leaves and stem.

The increasing interest in drug discovery from native medicinal plants has led to many studies on extraction, identification and quantification of bioactive compounds in different species of Pittosporum genus, *P. angustifolium* included [6,7,8,9,10,11,12,13,14]. Several bioactive compounds have been identified in the crude extracts of *P. angustifolium* such as triterpenoid saponins in leaves and seeds [7,8,9,10,11], phenolic acids and flavonoids in leaves (Figure 1) [12], tannins and essential oils in leaves and fruits [11]. Among them, triterpenoid saponins, essential oils and non-tannin polyphenols are reported as main bioactive compounds in the Pittosporum genus [6,13], whereas tannins and alkaloids are minor compounds. There seems to be no alkaloids present in the leaves and fruits of *P. angustifolium* [11,14].

Despite the wealth of available literature on bioactive compounds and their associated medicinal properties, there is still a gap in knowledge regarding potential effects of *P. angustifolium* bioactive compounds due to different growing conditions (wild vs. cultivated), diverse botanical tissues (leaf vs. stem) and geographical locations. Therefore, the present study aimed (i) to determine proximate composition, minerals and trace elements, bioactive compounds, antioxidant capacity and antimicrobial activity of Australian grown *P. angustifolium* as an initial measure of their nutritional value and bioactive potential and (ii) to assess the impact of different plant parts, growing conditions and geographic locations on bioactive compounds and associated bioactivities.

## 2. Materials and Methods

### 2.1. Plant Material

Approximately 5 kg of *P. angustifolium* (1 year-old) whole branch (length ≤ 30 cm; stem diameter < 5 mm) collected in 2018 from the field (Clermont, Queensland (QLD), Australia) were provided by Gumby Gumby Australia, Ltd. (Clermont, Australia). The cultivated sample was divided into 3 different parts: leaves, stems and the whole branch (without separation as used for traditional medicinal applications). The ratio between leaf and stem was 65–70:30–35 (*w/w*). The samples were freeze-dried at −50 °C for 48 h (CSK Climateck, CSK Scientific, Brisbane, Australia) and blended into a fine powder using a Waring Laboratory Blender (Australian Scientific, Australia).

The wild harvested sample, collected in 2018 from subtropical forest, included *P. angustifolium* whole branch from QLD (provided by Gumby Gumby Australia, Ltd.) and leaves from South Australia (SA) (supplied by Bush Food Australia, Ltd., Wilmington, Australia). After harvesting, the samples were air-dried indoors and blended into a fine powder (ca. 3 kg) as with the cultivated samples. The final (powdered) samples had a moisture content ≤5% and were stored at −35 °C for further analysis.

The following abbreviations were used to label the samples: QLD–Cul–WB, QLD–Cul–Leaf, QLD–Cul–Stem (cultivated *P. angustifolium* whole branch, leaf and stem samples collected in QLD); QLD–Wild–WB (*P. angustifolium* whole branch collected from the wild in QLD) and SA–Wild–Leaf (*P. angustifolium* leaves collected from the wild in SA).

### 2.2. Reagents

Polyphenol and carotenoid standards (HPLC grade), including (+/−) -catechin, gallic acid, rutin, isoquercetin, chlorogenic acid, caffeic acid, p-coumaric acid, ferulic acid, lutein, zeaxanthin and trans-beta-carotene, were purchased from Sigma-Aldrich (Castle Hill, NSW, Australia). Ascorbic acid, 2,2-diphenyl-1-picrylhydrazyl (DPPH), oleanolic acid and 1,4-dithiothreitol (DTT) were also from Sigma-Aldrich.

Pteroylmonoglutamic acid (PteGlu), tetrahydrofolate (H_4_folate), 5-methyltetrahydrofolate (5-CH_3_-H_4_folate), 5-formyltetrahydrofolate (5-CHO-H_4_folate), and their corresponding labeled isotopes were sourced from Merck Eprova (Schaffhausen, Switzerland).

Cultures of *Staphylococcus aureus* (strain 6571) and *Escherichia coli* (strain 9001) were obtained from the National Collection of Type Cultures (NCTC, Health Protection Agency Center for Infection, London, UK). *Candida albicans* (strain 90028) was sourced from the American Type Culture Collection (ATCC, In Vitro Technologies Pty, Ltd., Noble Park, Australia). Plate count agar and potato dextrose agar media (Thermo Fisher Scientific, Scoresby, Australia) were used to test antibacterial and antifungicidal activity.

### 2.3. Methods

#### 2.3.1. Proximate Analysis

Proximate analysis was performed at Symbio Alliance Laboratories (Eight Mile Plains, Australia), a National Association of Testing Authorities (NATA) accredited laboratory that complies with ISO/IEC 17,025:2005. The following NATA accredited in-house or standard AOAC methods were used: protein (AOAC method 990.03, [15]); total fat (AOAC method 991.36, [16]); saturated, mono-unsaturated, poly-unsaturated and trans fatty acids by gas chromatography with flame-ionization detector (in-house method CFH068.2); dry matter (AOAC method 934.01, [17]); ash content (AOAC method 942.05, [17]); total sugar, total dietary fiber and available carbohydrate by high performance liquid chromatography equipped with refractive index detector (in-house methods CFH001.1, CF057 and CF029.1). Proximate analysis was performed in duplicate including measurement of uncertainty.

#### 2.3.2. Mineral and Trace Element Analysis

Analysis of minerals (Ca, K, Mg, Na and P) was performed using inductively coupled plasma optical emission spectrometry (ICP-OES, Agilent 700, Agilent Technologies, Tokyo, Japan) after hot-block digestion. Analysis of trace elements was performed using inductively coupled plasma mass spectrometry (ICP-MS, Agilent 7700) after microwave digestion. The analysis was carried out at the Forensic and Scientific Services, Queensland, a NATA accredited laboratory. Details of the method have been described previously by Akter et al. [18].

#### 2.3.3. Analysis of Polyphenols

##### Extraction of Free Phenolic Compounds

Extraction of free polyphenols was carried out as reported previously [19], with modifications. Briefly, 200 mg powdered sample was homogenized with 80% methanol containing 0.1% HCl (*v/v*) using a vortex. The homogenate was subsequently placed in an ultrasonication bath for 30 min at room temperature (rt) to support the release of phenolic compounds, followed by centrifugation at 3900 rpm for 10 min at rt (Eppendorf Centrifuge 5804, Hamburg-Eppendorf, Germany). Supernatants were retained, while residues were re-extracted twice followed the procedure described above. The supernatants were combined and subjected to UHPLC-PDA-MS/MS analysis, DPPH radical-scavenging capacity assay and total phenolic content (TPC) measurement. The extraction was carried out in triplicate.

##### Extraction of Bound Phenolic Compounds

Extraction of bound phenolic compounds followed Adom and Liu [20] with modifications and details of the method were described previously by Phan et al. [21]. Briefly, following the free phenolic extraction, the residues were subjected to alkaline hydrolysis and subsequently extracted with ethyl acetate. The ethyl acetate extracts were dried at 40 °C under nitrogen flow and redissolved in 50% methanol containing 0.1% formic acid for further analysis.

##### U(H)PLC-PDA-MS/MS Analysis

Polyphenols were analyzed using a Waters Acquity^TM^ UPLC-PDA System (Waters, Rydalmere, Australia) with detailed chromatographic conditions summarized in Supplementary Table S1. Peak identities were confirmed using a Thermo high resolution Q Exactive mass spectrometer equipped with electrospray ionization (ESI) source and a Dionex Ultimate 3000 UHPLC system (Thermo Fisher Scientific Pty, Ltd., Scoresby, Australia). A full MS scan in negative ion mode was acquired from *m/z* 120 to 1000 at a resolving power of 70,000 full-width at half maximum. For the compounds of interest, a MS/MS scan range of *m/z* 100–1000 was selected, with normalized collision energy (NCE) at 35 V. The compound identification was based on comparing retention time, UV-Vis spectra, mass spectra and fragmentation patterns with those obtained from available standards and/or literature. Polyphenols were quantified at 320 nm, using external calibration curves of different polyphenol standards as stated in Section 2.2.

#### 2.3.4. Analysis of Carotenoids

##### Extraction

Carotenoid extraction followed the method previously reported by Djuikwo et al. [22] with slight modifications. Approximately, 100 mg powdered sample was homogenized with acetone and 95% ethanol containing 0.1% butylated hydroxytoluene (BHT) (*w/v*). Next, the samples were subjected to saponification for 30 min at rt using KOH (20% in methanol, *w/v*), while shaking at 100 rpm. Following the saponification, hexane/dichloromethane (DCM) mixture (70:30, *v/v*) containing 0.1% BHT was added to extract carotenoid compounds. NaCl 10% (*w/v*) was added to improve phase separation, followed by centrifugation at 3900 rpm for 5 min at rt. The upper hexane/DCM layer was collected, combined and then evaporated under nitrogen until dryness. The crude extract was freshly reconstituted in methanol/methyl tert-butyl ether (50:50, *v/v*) containing 0.1% BHT for UHPLC-PDA-MS/MS analysis. All procedures were performed on ice, under dim light, using amber glassware where possible. The extraction was performed in triplicate.

##### U(H)PLC-PDA-APCI-MS/MS Analysis

Carotenoids were analyzed using a Waters Acquity^TM^ UPLC-PDA system (Supplementary Table S1). Detected carotenoid compounds were identified using the same UHPLC-MS/MS system as described for the polyphenol analysis (Section 2.3.3) but employing an atmospheric pressure chemical ionization (APCI) operated in positive mode. A full MS scan (*m/z* 80–1200) was acquired. For the compounds of interest, a MS/MS scan range of *m/z* 80–650 was selected, with NCE at 20 V. Carotenoids were quantified at 450 nm, using external calibration curves of all-trans beta carotene, lutein and zeaxanthin. Concentrations of carotenoid standards were determined spectrophotometrically (Cintra 303, GBC Scientific Equipment, Braeside, Australia) using the specific molar absorption coefficients of carotenoids in solutions [23].

#### 2.3.5. Vitamins

##### Folates

Folates were analyzed by stable isotope dilution assay (SIDA) and UHPLC-PDA-MS/MS (Striegel et al. [24]). Briefly, 100 mg of powdered sample was extracted with MES buffer (pH 5). Labeled isotopic compounds (IS), including [^13^C_5_]-PteGlu, [^13^C_5_]-H_4_folate, [^13^C_5_]-5-CH_3_-H_4_folate and [^13^C_5_]-5-CHO-H_4_folate, were added at appropriated concentrations. A Shimadzu UHPLC-ESI-MS/MS system (Shimadzu Corp., Kyoto, Japan) equipped with a Shimadzu 8060 triple quadrupole mass spectrometer was employed (Supplementary Table S1). Multiple reaction monitoring (MRM) in positive mode was optimized to quantify individual folate vitamers and their corresponding labeled isotopes. External calibration curves for quantification of folate vitamers were constructed based on the ratios of peak areas of analytes vs. IS.

##### Vitamin C

Ascorbic acid (L-AA) extraction and analysis followed Campos et al. [25], with slight modifications. Briefly, 200 mg powdered sample was extracted with 3% meta-phosphoric acid containing 8% acetic acid and 1-mM EDTA. The reduction of dehydroascorbic acid (DHAA), which was also present in the extracts/samples, to L-AA was performed following the method of Spinola et al. [26]. Vitamin C (L-AA + DHAA) was determined using a Waters UPLC-PDA system (Supplementary Table S1) and an external calibration curve of L-AA at 245 nm was used for quantification.

##### Non-Folate B Vitamins

Analysis of vitamins B1, B2, B3, B5, B6, B7 and B12 was conducted at Symbio Alliance Laboratories, using NATA accredited in-house HPLC-PDA methods (CFH363, CFH364 and CFH366). The analysis were performed in duplicate including measurement of uncertainty.

#### 2.3.6. Total Phenolic Content

The free and bound polyphenol extracts were used for total phenolic content (TPC) measurement employing the Folin–Ciocâlteu reagent [27]. A microplate absorbance reader (Sunrise, Tecan, Maennedorf, Switzerland) was used at 700 nm. TPC was expressed as mg of gallic acid equivalents (GAE) per g of sample, using an external gallic acid standard curve (0–105 mg/L).

#### 2.3.7. DPPH Radical-Scavenging Capacity

The DPPH free radical-scavenging assay [28] was used to determine the radical-scavenging capacity of the samples. The scavenging capacity was measured on a microplate absorbance reader (Sunrise, Tecan) at 517 nm. Ascorbic acid (0–0.1 mg/mL) was added for comparison. The % DPPH scavenging capacity was calculated using the following equation:

% DPPH = [(A_0_ − A_1_)/A_0_] × 100;

where A_0_ and A_1_ are the absorbance values of the control and the test samples, respectively.

#### 2.3.8. Total (Condensed) Tannins

Extraction of tannins followed the method described by Karamac et al. [29] with slight modifications. Briefly, approximately 1 g powdered sample was extracted with acetone in a sonication bath (70 °C, 15 min). After cooling, the supernatant was retained by centrifugation at 3900 rpm for 10 min. The extraction was repeated 3 times and the supernatants were combined and concentrated at 40 °C in a miVac sample Duo concentrator (Genevac, Inc, Gardiner, NY, USA). The dried extract was redissolved in methanol and subjected to the Vanillin-HCl assay (Price et al. [30]), using a Sunrise microplate reader at 500 nm. (+/−)-Catechin (0–1.5 g/L) was used to prepare an external calibration curve. Total tannin content (TTC) was expressed as mg of catechin equivalents (CaE) per 100 g of sample.

#### 2.3.9. Total Saponins

Extraction and quantification of saponins followed the spectrophotometric method described previously [31,32] with modifications. Approximately 1 g powdered sample was extracted with 80% methanol at rt while shaking on an orbital shaker (RP1812, Paton Scientific, Victor Harbor, Australia) at 100 rpm overnight. The supernatant was collected after centrifugation (3900 rpm, 10 min), while the residue was re-extracted twice with 80% methanol (for 1 h). The supernatants were combined and evaporated until dryness at 40 °C in a miVac sample Duo concentrator. The dried extract was redissolved in water and successively extracted with diethyl-ether to remove the pigments, followed by extraction of saponins with saturated n-butanol. The n-butanol extracts were combined and dried under reduced pressure using a rotary evaporator (Buchi Rotavapor R-100, BÜCHI Labortechnik AG, Flawil, Switzerland). The dried extract was redissolved in aqueous methanol 80% (*v/v*) and subjected to the Vanillin-H_2_SO_4_ assay [31,32], using a Sunrise microplate reader at 540 nm. Oleanolic acid (0–0.5 g/L) was used to prepare an external calibration curve. Total saponins were expressed as mg of oleanolic acid equivalents (OE) per 100 g of sample.

#### 2.3.10. Antimicrobial Activity

Powdered samples (1 g) were extracted 3 times with water or methanol in a sonication bath (30 min, rt). The supernatants were combined after centrifugation and evaporated at 60 °C and 40 °C for water and methanolic extracts, respectively, using a miVac sample Duo concentrator. Aqueous methanol 20% (*v/v*) was used to freshly reconstitute the extract precipitates. Well diffusion assay followed the method described previously by Phan et al. [21] was applied to test the antimicrobial activity against *Staphylococcus aureus*, a Gram-positive bacteria; *Escherichia coli*, a Gram-negative bacteria and *Candida albicans*, a fungi. Penicillin and streptomycin (1 g) (Gibco, Life Technologies, Scoresby, Australia) and 10 μg fluconazole (Sigma-Aldrich) were used as antibacterial and antifungal controls, respectively. Aqueous methanol 20% (*v/v*) was also included in the assay to evaluate the effect of solvent on microbial growth. The agar plates were incubated at 37 °C for 24 h or 48 h (depending on growth), and the inhibition zones formed around the wells were recorded. The results were expressed as strong (>13 mm), moderate (6–12 mm), weak (≥5 mm) or no inhibitory activity (<5 mm) [33].

#### 2.3.11. Statistical Analysis

A one-way analysis of variance (ANOVA), using Minitab 17 for Windows (Minitab, Inc., State College, PA, USA), was employed to test the variances of measurements. A *p*-value of 0.05 or less was considered as statistically significant. Pearson’s correlation coefficient analysis was also applied to test correlations between bioactive compounds and bioactivities.

## 3. Results and Discussion

### 3.1. Proximate, Minerals and Trace Elements

#### 3.1.1. Proximate

The proximate results showed that the predominant nutritional components of *P. angustifolium* are carbohydrates and dietary fiber, followed by low amounts of protein and fat (Table 1). Comparing between the two growing conditions (wild vs. cultivated), protein and carbohydrate contents were higher in the cultivated sample (7.2% vs. 10.6% and 26.6% vs. 31.3%, respectively). In contrast, the wild sample contained more fat (4.6% vs. 3.4%) and ash (11.2% vs. 9.6%) than the cultivated one. The slight difference in the proximate composition probably reflects the potential effect of different growing conditions on the biosynthesis of nutritional components in *P. angustifolium*.

According to Food Standards Australia and New Zealand [34], if a serving of food contains at least 4 to 7 g of dietary fiber, it can be considered as a good to excellent source of dietary fiber. Based on this, *P. angustifolium* is a good source of dietary fiber (43.3–45.2% DW; Table 1) with a recommended serving size of 10 g powder per day (suggested by Gumby Gumby, Ltd.). Furthermore, a relative high content of ash could be determined (9.6–11.2% DW), indicating the presence of considerable concentrations of inorganic elements in this plant. Hence, it is necessary to determine minerals and trace elements to understand their nutritional significance in this plant.

#### 3.1.2. Minerals and Trace Elements

Generally, there was a significant (*p* < 0.05) difference in minerals and trace elements between *P. angustifolium* leaves and stems, with the leaves possessing significantly (*p* < 0.05) higher levels of minerals and trace elements than the stems (Table 2). Furthermore, the environment had a significant impact on minerals and chemical elements, particularly Ca, Na, K, P, Fe, Al, Ba, Hg and Pb (*p* < 0.05 between wild and cultivated samples). Only Mg and Ni were found at comparable levels in the wild and cultivated samples (*p* > 0.05). Several elements such as Ag, Sn and Sb were detected at relatively low levels (below 0.05 mg/kg DW), whereas Ca, K and Fe were found at relatively high levels which could be favorable in terms of their dietary intake (Table 2). For heavy metals, Al was found at the highest level among the heavy metals quantified, followed by Cr, Pb, Cd and Hg, with the Al content being found almost five times higher in the wild compared to the cultivated sample (89 vs. 18 mg/kg DW; Table 2). The obtained results suggest that the levels of minerals and trace elements in *P. angustifolium* are affected by different growing conditions as well as botanical tissues.

### 3.2. Vitamins

#### 3.2.1. Non-Folate B Vitamins

B vitamins are crucial in many metabolic and physiological processes and can act as coenzymes in the energy metabolism (vitamins B1, B2, B3, B5 and B7), production of new cells (vitamins B6 and B12), protein metabolism (vitamin B6), and are essential for a functioning nervous system (vitamins B1, B3 and B12) [40]. Table 3 shows that only vitamins B2, B3, B5 and B6 could be quantified in the *P. angustifolium* whole branch sample (59 to 1300 µg/100 g DW) and vitamins B1, B7 and B12 were only found in traces (<5 µg/100 g DW). Vitamin B3 was the highest among the analyzed B vitamins. Similar to the proximate composition, the cultivated sample contained more B vitamins (B2, B3 and B5) than the wild sample. Only vitamin B6 was higher in the wild sample. The recommended dietary intakes (RDI) for the analyzed B vitamins are also included in Table 3 for reference and extrapolation.

#### 3.2.2. Vitamin C

**Figure 2:**
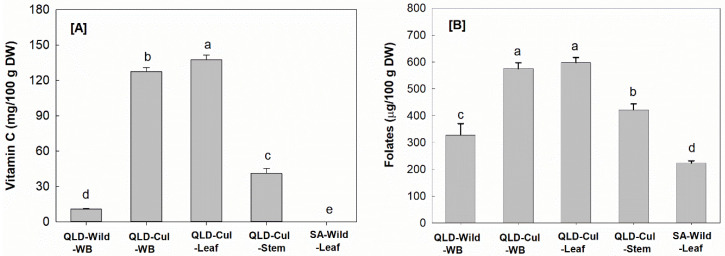
Contents of vitamin C (**A**) and folates (**B**) in the studied *P. angustifolium* samples; Data present means ± SD (*n* = 3); Different letters indicate significant differences at α = 0.05; RDI for vitamin C and folates are reported at 45 mg/day and 400 µg/day for adults, respectively [34].

Figure 2A shows that the cultivated leaf sample had the highest (*p* < 0.05) vitamin C content (137 mg/100 g DW) among the samples tested, followed by the whole branch and the stem samples. Ascorbic acid was not detectable (LOD = 0.1 ppm) in the *P. angustifolium* leaves collected from the wild in SA (SA–Wild–Leaf; Figure 2A). Regarding the impact of growing conditions, the wild samples had significantly (*p* < 0.05) lower vitamin C levels than the cultivated samples collected in QLD. The results reflect not only the importance of growing conditions and location, but also the drying procedure and sample treatment, which are critical in preserving vitamin C. The samples collected from the wild, both in SA and QLD, were air-dried, whereas the cultivated samples were freeze-dried, which is a very mild preservation procedure. Vitamin C found in the cultivated leaf sample was double the amount of that reported in green tea leaf powder (60 mg/100 g DW [41]).

#### 3.2.3. Folates

Folates were found at moderate levels in the *P. angustifolium* samples following the order: QLD–Cul–Leaf > QLD–Cul–WB > QLD–Cul–Stem > QLD–Wild–WB > SA–Wild–Leaf (Figure 2B). 5-CH_3_-H_4_folate, the biologically active folate form, was the main folate vitamer found in the studied *P. angustifolium* samples (data not shown). Among the analyzed tissues, the leaves had a significantly (*p* < 0.05) higher folate content than the stem, but no difference (*p* > 0.05) could be observed between the leaves and the whole branch samples. Similar to vitamin C (Figure 2A), the total folate content of the leaves collected in SA was the lowest (*p* < 0.05) among the samples studied (Figure 2B), indicating the impact of the geographic location and/or sample treatment. The results also showed that the levels of folate in *P. angustifolium* (222–597 µg/100 g DW, equivalent to 74–220 µg/100 g FW, considering a moisture content of 63%) are comparable to strawberry (59–153 µg/100 g FW [41]), a popular dietary source of folate. However, it should be noted that the common serving size of *P. angustifolium* (max. 10 g powder) is much lower than that of strawberry fruit (144 g).

### 3.3. Total Phenolic Content (TPC)

The TPC results (free and bound) ranged from 730 to 4075 mg GAE/100 g DW, depending on the plant tissue and geographic location, but was not affected by growing conditions (wild vs. cultivated) (Table 4). Furthermore, the free-TPC was considerably higher in all samples compared to the bound-TPC, as the free-TPC accounted for >90% of total TPC. The stems had lower total TPC (free and bound) compared to the leaf samples but contained more (*p* < 0.05) bound TPC than the leaves; however, the bound TPC only contributed a minor proportion to the total TPC.

There was no difference (*p* > 0.05) in the TPC of the samples collected from the wild or cultivated in QLD. In contrast, different growing locations affected the TPC, as the wild leaves collected in SA possessed ca. 3-fold more (*p* < 0.05) total phenolics than the QLD samples (Table 4). The results of the present study are similar to the reported TPC in leaves and twigs of other species belonging to the Pittosporaceae family, ranging from 2600 to 4090 mg GAE/100 g DW [42,43,44,45]. However, compared to green tea leaf powder, a popular source of polyphenols, the TPC in the studied *P. angustifolium* samples was lower (730–4075 mg GAE/100 g DW vs. 6500–10,600 mg/100 g DW [46]).

### 3.4. Total (Condensed) Tannins

The total tannin content (TTC) in the studied *P. angustifolium* samples ranged from 15.9 to 52 mg CaE/100 g DW (Table 4) and was much lower than the TPC. Furthermore, the TTC was significantly (*p* < 0.05) affected by the geographical location, whereas growing conditions and different plant tissues did not affect (*p* > 0.05) the TTC. The QLD-grown samples showed a significantly (*p* < 0.05) lower TTC compared to the SA-grown sample. There is also a controversy in reporting tannins in *Pittosporum* species, probably due to the diversity of genotypes. For example, Amoo et al. [44] reported that condensed tannins were not detectable in leaves and twigs of *P. viridiflorum* collected in KwaZulu-Natal (South Africa), whereas Vesoul and Cock [14] and Momeni et al. [47] reported the presence of tannins (qualitative data only) in *P. phylliraeoides* collected in Queensland (Australia) and *P. viridiflorum* grown in Western Cameroon (Africa), respectively.

### 3.5. Saponins

Table 4 shows that *P. angustifolium* leaves and stems are rich sources of saponins, with levels up to 4000 mg/100 g equivalent to 4% on dry weight basis. Comparison with common saponin-rich foods such as legume seeds, including chickpeas (230 mg/100 g DW), kidney beans (410 mg/100 g DW) and soya beans (650 mg/100 g DW) [48], the saponin content in the studied *P. angustifolium* samples is approximately 10-times higher. Contrary to the TTC and TPC, with SA leaves having significantly (*p* < 0.05) higher levels, the saponin content of *P. angustifolium* whole branch and leaves collected in QLD was higher than that of the SA leaves (trend only, *p* > 0.05; Table 4). Interestingly, the saponin level in the leaf sample was double that of the stem (3870 vs. 1589 mg OE/100 g DW), indicating a significant (*p* < 0.05) effect of the botanical tissue. There is limited information available regarding quantification of saponins in *P. angustifolium* as several previous studies only reported the presence of saponins in the *Pittosporum* genus by using qualitative screening tests without further quantification [11,14]. Recently, by applying NMR spectroscopy, mono- and bis-demosidic triterpene saponins as well as taraxastane-type triterpene saponins have been structurally identified as the main triterpenoid saponin compounds in *P. angustifolium* [8,9,10]. Further studies on profiling and quantification of individual saponins in this plant are necessary.

### 3.6. Individual Phenolic Compounds

**Figure 3:**
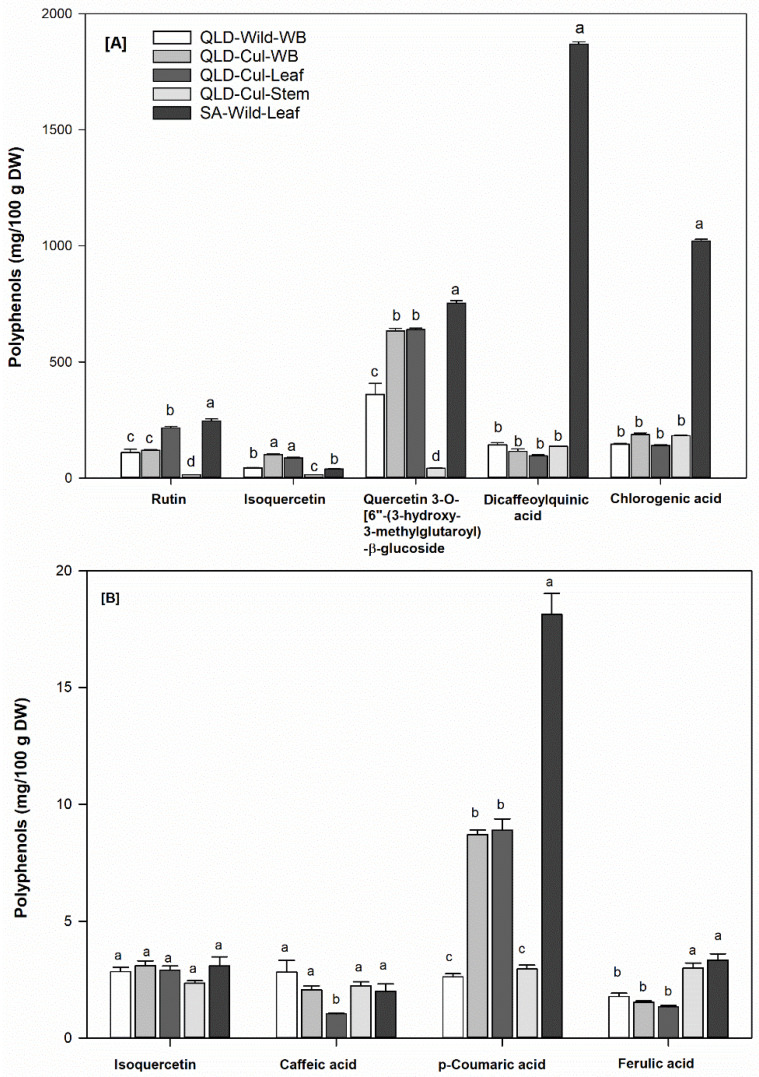
Free (**A**) and bound (**B**) phenolic compounds in the studied *P. angustifolium* samples; Data present means ± SD (*n* = 3); Different letters of individual phenolic compounds indicate significant differences at *α* = 0.05.

Figure 3A,B presents the predominant phenolic compounds identified in the studied *P. angustifolium* samples (further details are summarized in Supplementary Table S2 & Figure S1). In agreement with the TPC (Section 3.3), the total amount of free phenolic compounds was considerably higher than that of bound phenolics (689–3931 vs. 10.1–26.5 mg/100 g DW, data not shown), suggesting that the free polyphenols may be primarily responsible for potential biologic activities of *P. angustifolium*.

However, all samples showed a similar phenolic profile, the concentrations of individual phenolic compounds varied depending on the extract (free or bound), botanical tissues, growing condition and location. For instance, the free extract was rich in flavonoids, whereas phenolic acids were predominant in the bound extract (Figure 3). While the stem had significantly (*p* < 0.05) lower levels of free phenolic acids and flavonoids than the leaves, the leaves had lower levels of bound phenolic compounds compared to the stem, except for p-coumaric acid. Similar to the TPC, a significantly (*p* < 0.05) higher amount of phenolic acids and flavonoids (free > 3-fold and bound ca. 2-fold) was found in the SA leaves compared to the QLD grown samples (Figure 3), clearly indicating the effect of geographical location on individual polyphenols.

Previously, a number of studies have reported the presence of caffeic, ferulic and p-coumaric acids in methanolic extracts of *P. tobira* seeds [19] as well as rutin, isoquercitrin, isoquercitrin-hydroxymethylglutaroyl-β-glucoside and dicaffeoylquinic acids in the leaves of *P. angustifolium* [12] (Figure 1), which support the findings of the present study as shown in Figure 3A,B. Interestingly, chlorogenic acid, which has not been previously reported in *P. angustifolium*, was also detected in the studied samples (Figure 3A).

### 3.7. Carotenoids

**Figure 4:**
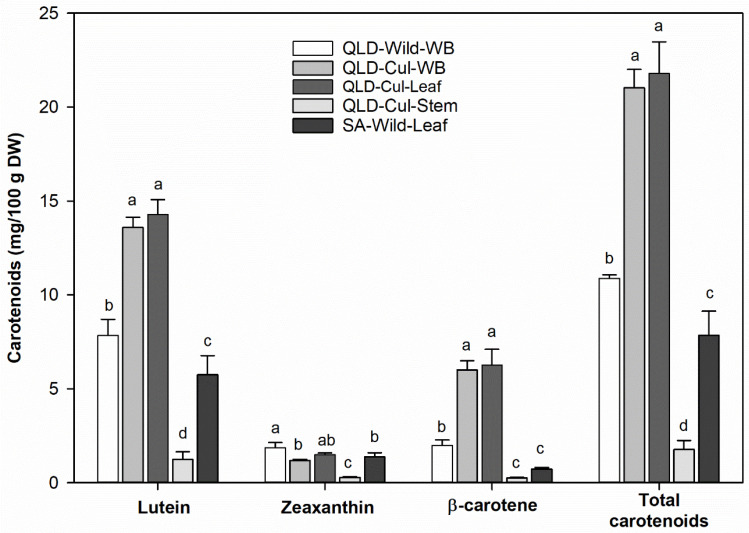
Carotenoids identified in the studied *P. angustifolium* samples. Data present means ± SD (*n* = 3); Different letters of individual carotenoid compounds indicate significant differences at *α* = 0.05.

Lutein, zeaxanthin and β-carotene could be identified as the main carotenoids in the *P. angustifolium* samples (Figure 4 and Supplementary Table S3 & Figure S2). Lutein was the predominant carotenoid (>70% of the total carotenoid content), followed by zeaxanthin or β-carotene (depending on the sample). The total carotenoid content varied from 1.8 to 21.8 mg/100 g DW, which is higher than that reported for sweet corn (3.7 mg/100 g DW [49]).

There was significant (*p* < 0.05) variation in carotenoid content between different botanical tissues. The stem had lower (*p* < 0.05) carotenoid levels than the leaves, suggesting that carotenoids are mainly synthesized and stored in the leaf tissue. An approximately 2-fold higher concentration of carotenoids (except for zeaxanthin) could be found in the cultivated sample compared to the wild samples collected from both QLD and SA. This is most likely due to differences in sample treatment and storage conditions (as also observed for vitamin C and folates). To the best of our knowledge, this is the first study reporting individual carotenoids in *P. angustifolium*.

### 3.8. DPPH Radical-Scavenging Capacity

**Figure 5:**
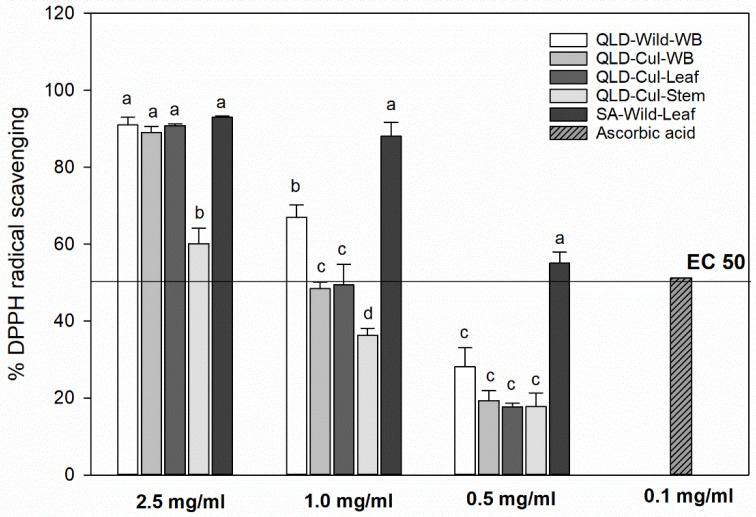
2,2-diphenyl-1-picrylhydrazyl (DPPH) radical-scavenging capacity of *P. angustifolium* extracts. EC_50_ value is the amount/concentration of the sample extract or ascorbic acid necessary to scavenge/reduce the initial DPPH radical concentration by 50% (50% effective concentration). Different letters within individual extract indicate significant differences at *α* = 0.05.

The leaf sample, collected from the wild in SA, exhibited a significantly (*p* < 0.05) higher DPPH radical-scavenging capacity at 0.5 and 1 mg powder/mL, followed by the QLD whole branch sample collected from the wild (QLD–Wild–WB) (Figure 5), which strongly correlates with the TPC and individual phenolic compounds. The cultivated samples showed significantly (*p* < 0.05) lower radical-scavenging capacity than the wild samples, except for the concentration of 2.5 mg powder/mL. The results also showed that the extracts from the SA leaves (0.5 g powder/mL) or from the QLD samples (0.7–1 g powder/mL) collected either from the wild or cultivated exhibited a similar DPPH radical-scavenging capacity (EC_50_) as 0.1 mg ascorbic acid (Figure 5). However, the cultivated leaf sample contained more vitamin C, folates and carotenoids than the samples collected from the wild (QLD and SA), more sample material was needed to reduce the initial DPPH radical concentration by 50% (EC_50_: 1 g powder/mL QLD cultivated leaves vs. 0.7 g powder/mL QLD wild leaves or 0.5-g powder/mL SA wild leaves). This clearly indicates a possible correlation of DPPH radical-scavenging capacity with polyphenols, as a result of their electron transfer/hydrogen donating ability. The SA leaves had the highest levels of TPC and tannins, resulting in the highest DPPH radical-scavenging capacity. The Pearson’s correlation coefficient test (Section 3.10) supports this interpretation.

### 3.9. Antimicrobial Susceptibility Test

Both methanolic and water extracts of the *P. angustifolium* samples showed inhibition against *C. albicans*, with inhibition zones being similar to the standard antibiotic solution (Table 5). However, no inhibition against *E. coli* and *S. aureus* was observed, except for the SA leaves which showed a moderate inhibition to *S. aureus*. The observed inhibition zones varied from 12.8 to 26.9 mm, suggesting a strong antimicrobial activity of the extracts from *P. angustifolium* against *C. albicans*. Although there was a substantial variation in bioactive compounds, depending on the botanical tissue, growing condition and geographical location, comparable antifungal activity was observed for all samples, except for the SA leaves, which showed higher antimicrobial activity (trend only, *p* > 0.05) compared to the QLD grown samples (Table 5). Furthermore, the water extract of the QLD whole branch sample collected from the wild showed significantly lower (nearly 2-fold; *p* < 0.05) inhibition compared to the cultivated sample, although those samples had similar levels of bioactive compounds. This suggests that the microbial inhibitory effect may be primarily driven by a synergistic effect of the polyphenol/bioactive compounds “complex” present in the extracts rather than a specific subclass or individual bioactive compound as previously hypothesized by Barbieri et al. [50].

A comparison between the two extraction methods showed that the water extract exhibited stronger antifungal activity than the methanolic extract, except for the QLD–Wild–WB sample (Table 5). Differences in the antimicrobial activity between the two extraction methods may be due to differences in the extraction efficiency resulting in higher or lower concentrations of specific bioactive compounds with antimicrobial activity (e.g., against *C. albicans*). The obtained results of antimicrobial activity are in agreement with the study of Vesoul and Cock [14], who previously reported a relatively low or no inhibitory effect of water and/or methanolic extracts of *P. phylliraeoides* against *E. coli* and *S. aureus*.

### 3.10. Pearson’s Correlation Analysis

The results of Pearson’s correlation test (Table 6) indicated a positive correlation (R^2^ = 0.64–0.92, *p* < 0.05) between polyphenols (e.g., TPC, rutin, quercetin 3-O-[6”-(3-hydroxy-3-methylglutaroyl)-β-glucoside], dicaffeoylquinic acid and tannins) and the DPPH radical-scavenging capacity. Saponins and polyphenols, particularly tannins, rutin and quercetin 3-O-[6″-(3-hydroxy-3-methylglutaroyl)-β-glucoside], one of the main phenolic compounds present in the free form, showed a positive (R^2^ = 0.76–0.81, *p* < 0.05) correlation with the antifungal activity. However, vitamin C (another strong antioxidant), folates and carotenoids were negatively correlated with the DPPH radical-scavenging capacity and antimicrobial activity (Table 6). This may be due to the relatively low concentrations of these vitamins and pro-vitamins in the tested samples, which were most likely too low to show any significant effects in reducing the DPPH radicals or antimicrobial activity. The results of the Pearson’s correlation test provided a general understanding on how bioactive compounds may contribute to the observed biologic effects of *P. angustifolium*.

## 4. Conclusions

The present study, to the best of our knowledge, reports for the first time the proximate composition, minerals and trace elements, vitamins and carotenoids in *P. angustifolium* leaves and stems collected from the wild or cultivated. *P. angustifolium* leaves could be identified as a rich source of saponins and polyphenols, whereas carotenoids, tannins and vitamins (B and C) were present at lower levels. The results indicate that multiple factors, such as growing condition, geographic location and different botanical tissues, can have a significant effect on the bioactive compounds in *P. angustifolium* and subsequently its bioactivity. However, further studies with more samples (total number and replicates), different seasons and growing locations, are strongly recommended to substantiate the results of the present study. The finding of the Pearson’s correlation suggests that only polyphenols have a significant correlation with the DPPH radical-scavenging capacity, whereas the antifungal activity against *C. albicans* was positively correlated with both polyphenols and saponins. This study further confirms the relationship between (phyto) chemicals and biologic properties in *P. angustifolium*, suggesting potential applications of this Australian indigenous plant as a functional (food) ingredient and/or a natural fungicide.

## Figures and Tables

**Table 1:** Proximate composition of *P. angustifolium* whole branch samples collected in Queensland.

Proximate Composition		QLD_Wild_WB	QLD_Cul_WB
		Quantity (g/100 g DW) *	
**Protein**		7.2	10.6
**Fat**	Total fat content	4.6	3.4
	Saturated fatty acids	1.8	0.9
	Monounsaturated fatty acids	0.4	0.2
	Polyunsaturated fatty acids	2.4	2.3
	Trans fatty acids	<0.01	<0.01
**Carbohydrate**	Total carbohydrate	26.6	31.3
	Soluble sugars	6.3	9.5
**Total dietary fiber**		45.2	43.3
**Moisture**		5.2	1.7
**Dry matter**		94.8	98.3
**Ash**		11.2	9.6

* Data present means of duplicate analysis with a measurement of uncertainty below 10%; DW—dry weight.

**Table 2:** Minerals and trace elements of different *P. angustifolium* samples collected in Queensland.

Chemical Elements		QLD–Wild–WB	QLD–Cul–WB	QLD–Cul–Leaf	QLD–Cul–Stem	Nutritional Information
		mg/kg DW *				
Minerals	Ca	27,302 ± 190 a	7879 ± 99 c	10,095 ± 31 b	3295 ± 194 d	1200 mg/day AI [35]
	K	15,403 ± 8 d	22,766 ± 179 b	26,180 ± 120 a	19,131 ± 528 c	4.7 g/day AI [35]
	Mg	2547 ± 9 b	2590 ± 21 b	3284 ± 10 a	989 ± 43 c	350 mg/day EAR [35]
	Na	878 ± 3 c	1161 ± 5 b	2083 ± 15 a	686 ± 19 d	1300 mg/day AI [35]
	P	1194 ± 14 d	2669 ± 31 b	2844 ± 17 a	2286 ± 37 c	700 mg/day AI [35]
Trace elements	B	68 ± 1.0 a	48 ± 0.6 c	57.7 ± 0.6 b	16.7 ± 1.0 d	–
	Al	89 ± 4.4 a	18 ± 0.6 c	28.3 ± 0.2 b	7.5 ± 0.1 d	1.0 mg/kg BW/week TWI [36]
	V **	0.14 a	0.04 c	0.05 b	0.02 d	–
	Cr **	0.33 a	0.11 b	0.16 b	0.14 b	35 µg/day AI [35]
	Mn	41.7 ± 0.6 b	39.7 ± 0.6 c	60.3 ± 0.6 a	12.3 ± 0.6 d	2.3 mg/day AI [35]
	Fe	116.7 ± 5.8 a	48.3 ± 1.0 b	56 ± 0.1 b	17 ± 1.2 c	8 mg/day RDA [35]
	Co **	0.1 a	0.05 b	0.05 b	0.02 c	–
	Ni	0.9 ± 0.1 b	1.1 ± 0.1 b	1.57 ± 0.1 a	1 ± 0.1 b	–
	Cu	8.1 ± 0.1 b	8.9 ± 0.1 a	6.8 ± 0.1 c	7.6 ± 0.2 d	900 µg/day AI [35]
	Zn	57 ± 1.0 c	62 ± 0.1 b	76.7 ± 1.2 a	19.7 ± 1.0 d	11 mg/day RDA [35]
	As **	0.03 c	0.04 b	0.06 a	0.02 d	128 µg/week for a 60 kg BW TWI [37]
	Se **	0.06 b	0.08 a	0.08 a	0.04 c	55 µg/day AI [35]
	Sr	140 ± 0.1 a	47.3 ± 0.6 b	57.7 ± 0.4 b	32 ± 0.6 d	–
	Mo **	0.2 c	0.7 b	0.8 a	0.2 c	45 µg/day AI [35]
	Ag	<0.01	<0.01	<0.01	<0.01	–
	Cd **	0.05 c	0.06 b	0.07 a	0.06 b	2.5 µg/kg BW/week TWI [38]
	Sn	<0.05	<0.05	<0.05	<0.05	–
	Sb	<0.01	<0.01	<0.01	<0.01	–
	Ba	36 ± 0.3 a	3.3 ± 0.1 c	4.0 ± 0.2 b	3.4 ± 0.1 c	–
	Hg **	0.03 b	0.05 a	0.01 c	<0.01	5 µg/kg BW/week TWI [39]
	Pb **	0.12 a	0.06 c	0.08 bc	0.09 b	25 µg/kg BW/week TWI [39]

* Data present means ± SD (*n* = 3); ** SD ≤ 0.01; Different letters at the same row indicate significant differences among the samples at α = 0.05; DW—dry weight; (-)—not available; RDA—recommended dietary allowance, AI—adequate intake, EAR—estimated average requirement; BW—body weight; TWI—tolerable weekly intake.

**Table 3:** Selected B vitamins in *P. angustifolium* whole branch samples collected in Queensland.

Vitamin	QLD–Wild–WB	QLD–Cul–WB	Nutrition Information [34]
	Quantity (per 100 g DW) *		
B1 (Thiamin)	<5 µg	<5 µg	1.1–1.2 mg/day RDI
B2 (Riboflavin)	<5 µg	73 µg	1.6 mg/day RDI
B3 (Niacin)	840 µg	1300 µg	14–16 mg/day RDI
B5 (Pantothenic acid)	<5 µg	300 µg	4–6 mg/day RDI
B6 (Pyridoxine)	180 µg	59 µg	1.7 mg/day RDI
B7 (Biotin)	<5 µg	<5 µg	25–30 µg/day RDI
B12 (Cyanocobalamin)	<5 µg	<5 µg	2.4 µg/day RDI

* Data present means of duplicate analysis with a measurement of uncertainty below 10%; DW—dry weight; RDI—recommended dietary intake for adults;

**Table 4:** TPC, tannins and saponins in the studied *P. angustifolium* samples.

Samples	TPC (mg GAE/100 g DW)			Total (Condensed) Tannins (mg CaE/100 g DW)	Total Saponins (mg OE/100 g DW)
	Free	Bound	Total Free and Bound		
QLD_Wild_WB	1306 ± 41 b	44.2 ± 7.7 c	1350 ± 47 b	32 ± 1.2 b	3645 ± 351 ab
QLD_Cul_WB	1177 ± 110 b	44.6 ± 2 c	1222 ± 112 b	20 ± 2.6 bc	2869 ± 343 b
QLD_Cul_Leaf	1254 ± 59 b	40.9 ± 1.4 c	1295 ± 60 b	26.1 ± 6.6 bc	3871 ± 157 a
QLD_Cul_Stem	653 ± 31 c	76.3 ± 7.8 b	730 ± 27 c	15.9 ± 3.2 c	1590 ± 223 c
SA_Wild_Leaf	3985 ± 103 a	90.9 ± 4.2 a	4075 ± 99 a	52 ± 6.4 a	3250 ± 319 ab

Data present means ± SD (*n*= 3). Different letters at the same column indicate significant differences at *α* = 0.05.

**Table 5:** Antimicrobial activity of the studied *P. angustifolium* samples.

Samples	*E. coli*		*S. aureus*		*C. albicans*	
	Water Extraction	MeOH Extraction	Water Extraction	MeOH Extraction	Water Extraction	MeOH Extraction
	Inhibition Zone (mm) *					
QLD–Wild–WB	–	–	–	–	13.6 ± 2.2 c	19.5 ± 1.7 bc
QLD–Cul–WB	–	–	–	–	24.7 ± 0.7 ab	20.6 ± 0.6 ab
QLD–Cul–Leaf	–	–	–	–	23.1 ± 1.6 ab	21.2 ± 1.9 ab
QLD–Cul–Stem	–	–	–	–	24.0 ± 0.8 ab	21.0 ± 1.8 ab
SA–Wild–Leaf	–	-	12.8 ± 0.9	–	26.9 ± 1.6 a	25.3 ± 2.4 ab
Antibiotic/antifungal control **	29.2		55.8		27.1	

(*) Data present means ± SD (*n* = 3); (**) antibiotic and antifungal control as stated in the method section; (-) No inhibition zone was observed. Different letters indicate significant differences among the samples at α = 0.05.

**Table 6:** Pearson’s correlation coefficients between bioactive compounds, DPPH radical-scavenging capacity and antifungal activity.

Bioactive Compounds	% DPPH Free Radical-Scavenging			*C. albicans* Inhibition	
	2.5 mg/mL	1 mg/mL	0.5 mg/mL	Water Extract (0.5 mg/mL)	MeOH Extract (0.5 mg/mL)
TPC	0.78 **	0.87 ***	0.92 ***	0.24	0.76 *
Rutin	0.76 **	0.69 *	0.66 *	0.57	0.64
Isoquercetin	−0.03	−0.40	−0.18	−0.48	−0.05
Quercetin 3-O-[6″-(3-hydroxy-3-methylglutaroyl)-β-glucoside]	0.71 **	0.65 *	0.64 *	0.60	0.81 *
Dicaffeoylquinic acid	0.48	0.58	0.60 *	0.48	0.36
Chlorogenic acid	0.36	0.45	0.41	0.57	0.48
Vitamin C	−0.58 *	−0.65 *	−0.73 **	−0.32	−0.42
Folates	−0.59 *	−0.68 *	−0.64 *	−0.36	−0.36
Carotenoids	−0.07	−0.10	−0.21	−0.55	0.048
Saponins	0.26	0.37	0.26	0.79 *	0.31
Tannins	0.75 **	0.92 ***	0.83 ***	0.38	0.55

Statistical significance at *—*p* < 0.05, **—*p* < 0.01 and ***—*p* < 0.001.

## Supplementary Materials

The following are available online at https://www.mdpi.com/2304-8158/9/7/887/s1: Figure S1: Representative ion chromatograms and fragmentation patterns of detected phenolic compounds in *P. angustifolium*. Figure S2: Representative ion chromatograms and fragmentation patterns of detected carotenoids in *P. angustifolium*. Table S1: Details of chromatographic analysis of carotenoids, polyphenols, ascorbic acid and folates [51]. Table S2: Characterization of phenolic compounds detected in *P. angustifolium* by UHPLC-ESI-MS/MS scanning at negative mode. Table S3: Characterization of carotenoid compounds detected in *P. angustifolium* by UHPLC-APCI-MS/MS scanning at positive mode.
