# Supplementary material for: Nutritional Value and Antimicrobial Activity of Pittosporum angustifolium (Gumby Gumby), an Australian Indigenous Plant
Source: Foods. 2020 Jul 6;9(7):887. doi: 10.3390/foods9070887 (PMC7404462; doi:10.3390/foods9070887)
Supplement: Supplementary file 1 [file foods-09-00887-s001.zip › Supplementary files/Supplementary Table S1.docx]

**Table 1. Detail chromatographic analysis of carotenoids, polyphenols, ascorbic acid and folates**

| Bioactive compounds | Analytical Instrument | Stationary phase | Mobile phase | Gradient program | Reference |
| --- | --- | --- | --- | --- | --- |
| Carotenoids | A Waters Acquity^TM^ UPLC–PDA System (Waters, Milford, MA USA). The detection signals were recorded in the range 200-500 nm. Empower^TM^ software (Waters) was employed for process of peak areas and quantification. | A YCM C30 carotenoid column (3.6 x 250 mm, 3.6 µm) (Waters, Milford, MA, USA) maintained at 25 °C. | A: 0.1% formic acid in methanol (v/v)  B: 0.1% formic acid in MTBE (v/v) | The gradient program of mobile phase B was as follows: (20% B, 0 min), (25% B, 20 min), (30% B, 30 min), (70% B, 33 min), followed by 6 min equilibration to the initial condition  Injection volume: 5 µL  Flow rate: 0.6 mL/min | O’Hare*, et al*. [49] (with slight modifications) |
| Polyphenols |  | A Waters HSS-T3 column (100 x 2.1 mm i.d; 1.8 μm) (Waters, Milford, MA, USA) maintained at 40°C. | A: 0.1% aqueous formic acid  B: 0.1% formic acid in acetonitrile (v/v) | The gradient program of mobile phase B was as follows: (5% B, 0 min), (20% B; 3 min), (20% B, 4.3 min), (45% B; 9 min), (100% B, 11 min), followed by 6 min equilibration to the initial condition.  Injection volume: 2 µL  Flow rate: 0.4 mL/min | Gasperotti, *et al*. [51] |
| Ascorbic acid |  | A Waters HSS-T3 column (100 x 2.1 mm i.d; 1.8 μm) (Waters, Milford, MA, USA) maintained at 35°C. | 0.1% aqueous formic acid | Isocratic condition  Injection volume: 2 µL  Flow rate: 0.3 mL/min | Spinola, *et al*. [26] |
| Folates | A Shimadzu UHPLC-ESI-MSMS system (Shimadzu Corp., Kyoto, Japan) equipped with a Shimadzu 8060 triple-stage quadrupole mass spectrometer. | A Raptor ARC-18 column (2.7 µm, 100 × 2.1 mm) (Restek, Bad Homburg, Germany) maintained at 30°C. | A: 0.1% aqueous formic acid  B: 0.1% formic acid in acetonitrile (v/v) | The gradient program of mobile phase B was as follows: (3% B, 1 min), (10% B; 2.5 min), (10% B; 5 min), (15% B, 10 min), (50% B, 12 min) followed by 6 min equilibration to the initial condition.  Injection volume: 10 µL  Flow rate: 0.4 mL/min | Striegel, *et al*. [24] |
