# Supplementary material for: Nutritional Value and Antimicrobial Activity of Pittosporum angustifolium (Gumby Gumby), an Australian Indigenous Plant
Source: Foods. 2020 Jul 6;9(7):887. doi: 10.3390/foods9070887 (PMC7404462; doi:10.3390/foods9070887)
Supplement: Supplementary file 1 [file foods-09-00887-s001.zip › Supplementary files/Supplementary Table S2.docx]

**Supplementary Table S2. Characterization of phenolic compounds detected in *P. angustifolium* by UHPLC-ESI-MS/MS scanning at negative mode.**

| **Tentative identification** | **Retention Time**  **(min)** | **Molecular ion**  **[M-H]^-^** | **UV max (nm)** | **Fragments** | **References** |
| --- | --- | --- | --- | --- | --- |
| Caffeic acid | 3.8 | 179.0438 | 295/325 | 179.0438 | Bäcker, *et al*. [12] |
| Chlorogenic acid | 4.1 | 353.0873 | 303/326 | 191.0547 |  |
| p-Coumaric acid | 4.6 | 163.0401 | 227/310 | 163.0401 |  |
| Ferulic acid | 5.1 | 193.0495 | 235/323 | 193.0495 |  |
| Rutin | 6.0 | 609.1457 | 256/354 | 301.0496 |  |
| Isoquercetin | 6.2 | 463.0881 | 256/353 | 301.0496 |  |
| Quercetin 3-O-[6’’-(3-hydroxy-3-methylglutaroyl)-β-glucoside] | 6.8 | 607.1219 | 256/354 | 463.0881, 301.0496 |  |
| Dicaffeoylquinic acid isomers | 6.9/7.4 | 515.1191 | 236/325 | 351.1087, 191.551 |  |
