# Supplementary material for: Nutritional Value and Antimicrobial Activity of Pittosporum angustifolium (Gumby Gumby), an Australian Indigenous Plant
Source: Foods. 2020 Jul 6;9(7):887. doi: 10.3390/foods9070887 (PMC7404462; doi:10.3390/foods9070887)
Supplement: Supplementary file 1 [file foods-09-00887-s001.zip › Supplementary files/Supplementary Table S3.docx]

**Supplementary Table S3: Characterization of carotenoid compounds detected in *P. angustifolium* by UHPLC-APCI-MS/MS scanning at positive mode.**

| Tentative identification | Retention time  (min) | Molecular ion  [M+H]^+^ | Fragments | UV max (nm) | Reference |
| --- | --- | --- | --- | --- | --- |
| Lutein | 11.03 | 569.4344 | 551.4251, 533.4145 459.3620, 431.3306, 177.1844 | 446/474 | Rivera, *et al*. [51] |
| Zeaxanthin | 12.77 | 569.4344 | 551.4251, 533.4145 476.3647, 337.2523, 175.1482 | 449/476 |  |
| 13-cis-beta-carotene | 25.8 | 537.4454 | 413.3201, 321.2575, 177.1851, 137.1325 | 445/473 |  |
| All-trans-beta-carotene | 31.13 | 537.4454 | 481.3826, 413.3201, 177.1852, 137.1325 | 452/478 |  |
| 9-cis-beta-carotene | 32.37 | 537.4454 | 413.3201, 321.2575, 177.1851, 137.1325 | 445/473 |  |
