# Supplementary figures and images for: Nutritional Value and Antimicrobial Activity of Pittosporum angustifolium (Gumby Gumby), an Australian Indigenous Plant
Source: Foods. 2020 Jul 6;9(7):887. doi: 10.3390/foods9070887 (PMC7404462; doi:10.3390/foods9070887)

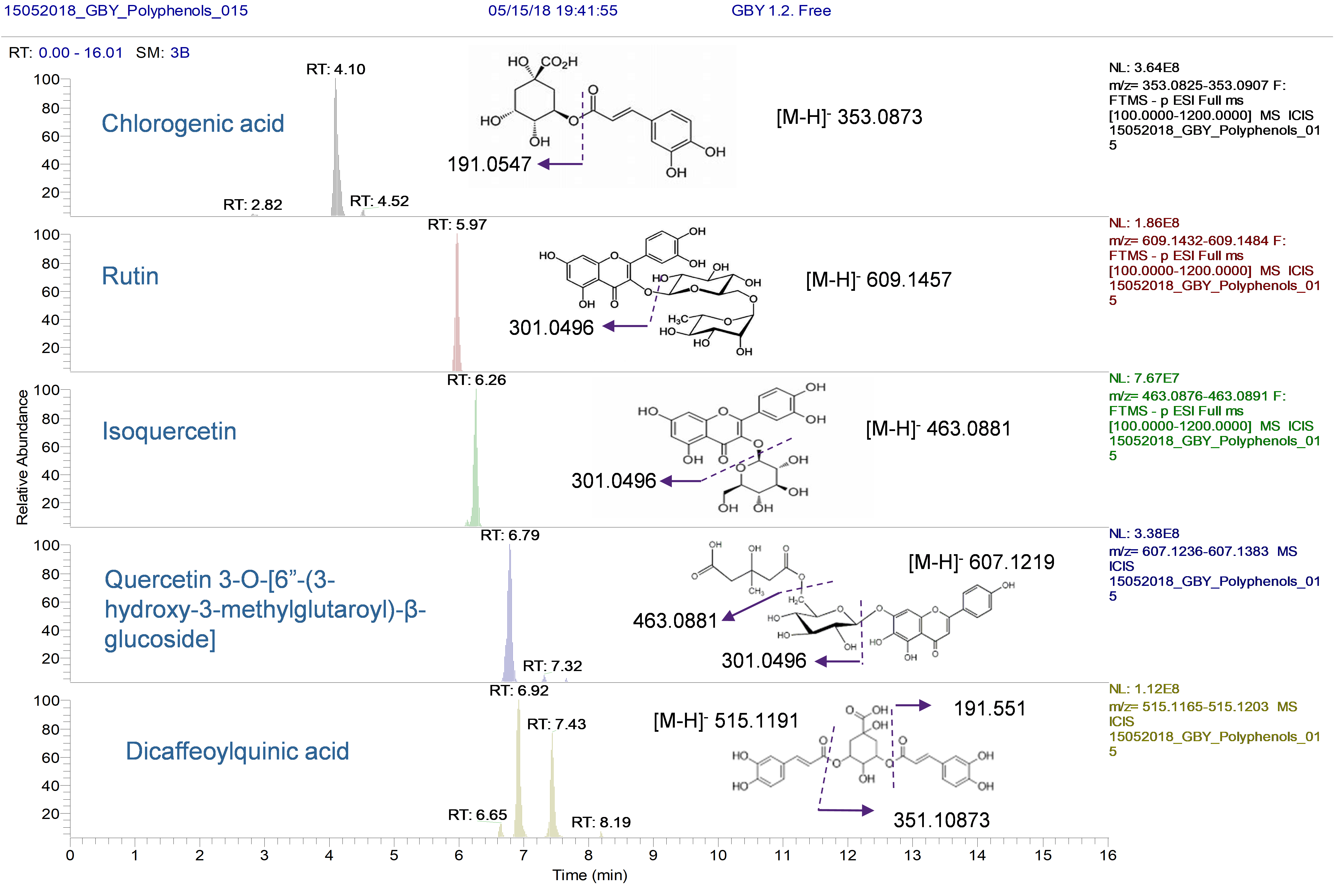

Supplement: Supplementary file 1 [file foods-09-00887-s001.zip › Supplementary files/Supplementary Figure S1_tiff.tif]

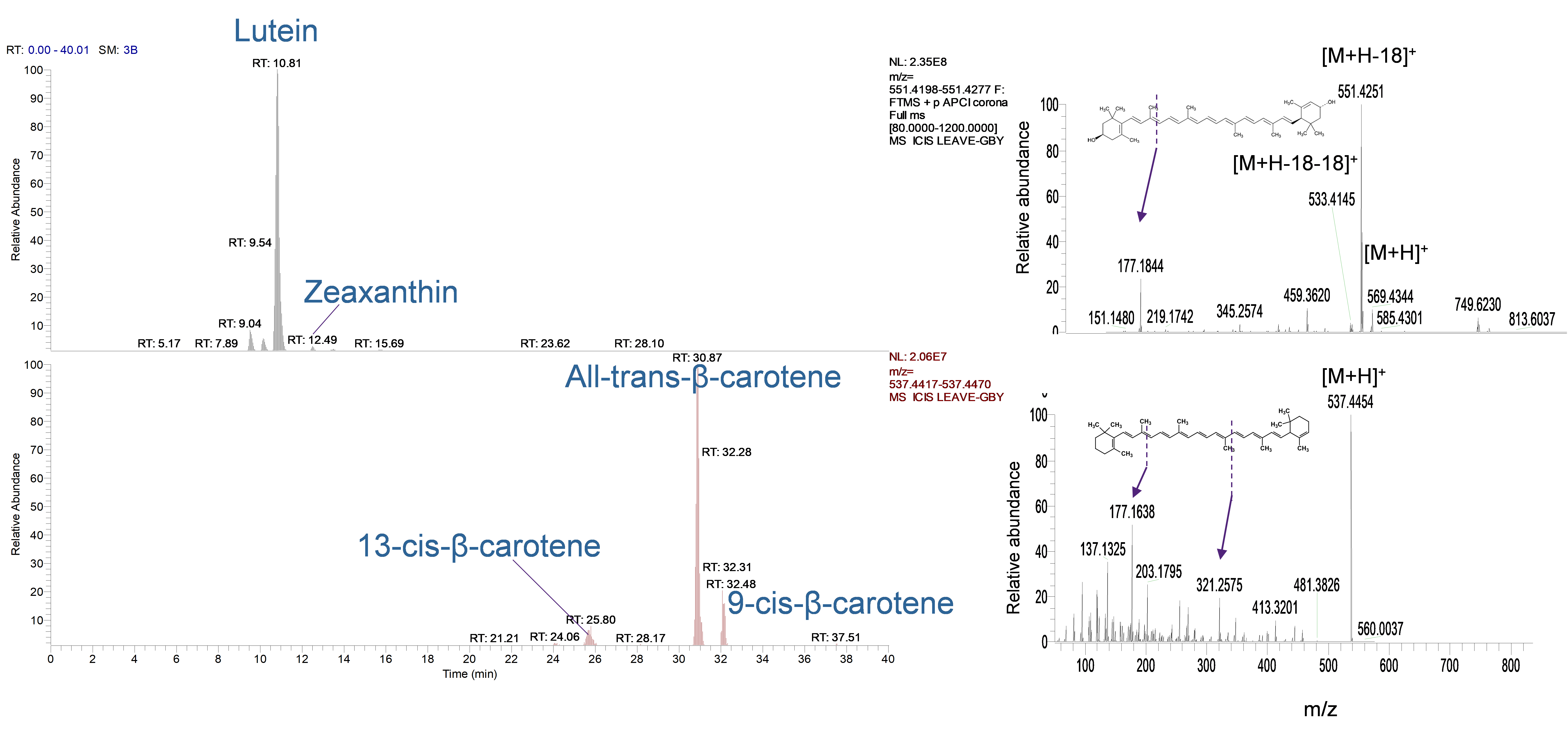

Supplement: Supplementary file 1 [file foods-09-00887-s001.zip › Supplementary files/Supplementary Figure S2_tiff.tif]
